# Efficacy of pillar implants to reduce snoring and daytime sleepiness

**DOI:** 10.2144/fsoa-2021-0020

**Published:** 2021-03-29

**Authors:** Laith Khasawneh, Haitham Odat, Basheer Y Khassawneh, Khalid A Kheirallah, Adi H Khassawneh, Ahmad Al Omari, Maisa Smadi, Firas Alzoubi, Safwan Alomari, Abdel-Hameed Al-Mistarehi

**Affiliations:** 1 Department of Surgery, Faculty of Medicine, The Hashemite University, Zarqa 13133, Jordan; 2 Department of Special Surgery, Faculty of Medicine, Jordan University of Science & Technology, Irbid, 22110, Jordan; 3 Department of Internal Medicine, Faculty of Medicine, Jordan University of Science & Technology, Irbid, 22110, Jordan; 4 Department of Public Health & Family Medicine, Faculty of Medicine, Jordan University of Science & Technology, Irbid, 22110, Jordan; 5 Department of Neurosurgery, Johns Hopkins University School of Medicine, Baltimore, MD, 21287, USA

**Keywords:** daytime sleepiness, ESS, pillar implants, snoring, VAS

## Abstract

**Objective:**

To measure the efficacy of pillar implants in reducing snoring.

**Materials and methods:**

A total of 30 adult patients who underwent pillar implants were assessed preoperatively and at 1, 3, 6 and 12 months after the implantation. Improvement was measured using snoring frequency, visual analog scale for snoring loudness, and Epworth sleepiness scale for daytime sleepiness.

**Results:**

The mean snoring frequency, loudness and Epworth score were reduced from 6.9, 9.2 and 7.4 at the baseline to 5, 5.9 and 5.6, respectively, at 12 months postoperatively (all p < 0.03). The partial implant extrusion rate was 6.7%.

**Conclusion:**

We suggest that a pillar implant procedure should be considered before proceeding to more morbid surgeries in patients with snoring and daytime sleepiness.

## Lay abstract

This is a study of 30 adult patients who underwent pillar implants for treating socially unacceptable snoring and, to a lesser extent, daytime sleepiness. We found that snoring frequency and loudness significantly improved at 1 month after the operation and that the improvement persisted until the end of the follow-up visits. Daytime sleepiness improvement was subjective at 1 month but statistically significant at 12 months postoperatively (p < 0.01). We conclude that the pillar implant technique leads to a significant reduction in snoring and daytime sleepiness, thus improving patients’ quality of life. It has very low morbidity.

Snoring is a condition in which there is increased resistance to airflow in the upper respiratory tract during sleep, resulting in loud sounds [1–3]. Snoring for three or more nights per week is termed habitual snoring and is mostly related to obstructive sleep apnea (OSA) and hypopnea [4–7]. However, it can also be a standalone problem in many patients [2,8,9].

Daytime sleepiness or excessive sleepiness is a serious symptom of some other conditions [10–12]. Patients with daytime sleepiness often complain of feeling drowsy and sluggish all the time, and sleep attacks when it is not desired [10,12]. This is mainly caused due to poor sleep habits, irregular sleep schedules, sleep disorders such as OSA, narcolepsy, hypersomnia, insomnia and periodic limb movements of sleep, adverse reactions from certain drugs, and other underlying medical conditions [10,12,13]. This condition interferes with routine activities, affecting patients’ productivity and relationships [14–16].

Snoring and daytime sleepiness are common problems worldwide, affecting 20% of the adult population [17,18]. Snoring has a tremendous social impact and causes severe social embarrassment and disharmony and discord with sleep partners; it also frequently leads patients to seek treatment [17,19–21]. Excessive daytime sleepiness is one of the most common sleep-related problems in patients with OSA [22]. It can significantly negatively impact performance, health and safety, and such patients are often perceived as lazy or unmotivated [23–25]. Therefore proper management of excessive daytime sleepiness is of paramount importance to improve quality of life.

Objective assessment of daytime sleepiness is expensive and time-consuming and needs a sleep laboratory. Many researchers have used excessive daytime sleepiness to assess the responsiveness of OSA to specific treatments [26,27]. The Epworth sleepiness scale (ESS) is a simple, inexpensive and easily administered subjective tool used to assess daytime sleepiness. Possible scores ranges from 0 to 24; the higher the score, the higher the daytime sleepiness [28].

Although several nonsurgical and surgical methods are used to manage snoring and OSA, patients should be compliant and accept the morbidity associated with these management methods [9,29]. There are treatment options for snoring and daytime sleepiness, including lifestyle modifications (avoiding risk factors, sleep position training), surgery, appliances (nasal dilators) and continuous positive airway pressure (CPAP) appliances. [9]. CPAP is a treatment of choice for sleep apnea [30–32]. Some surgical procedures that can help reduce snoring are tonsillectomy and adenoidectomy, septoplasty, uvulopalatopharyngoplasty (UPPP), somnoplasty and laser-assisted uvulopalatoplasty (LAUP) [9,33,34]. However, there is no optimal treatment for primary snoring to achieve long-term effectiveness while being minimally invasive [35].

Given that vibrations of the soft palate structure create the snoring sound, strengthening the soft palate could reduce the vibrations and snore [20,21,35,36]. In 2003 a pillar implant was introduced for treating snoring and mild/moderate OSA; it strengthened the soft palate and thus reduced the airways’ vibrations. Inserting the implants is a simple, easy and reversible office procedure with minimal morbidity and complication rate. In this procedure, three small (18 mm) polyethylene terephthalate rods are inserted in the soft palate mucosal layer sagittally, parallel to each other and spaced 2 mm apart to strengthen them by causing fibrosis and scarring through induction of reaction toward foreign bodies [17,19,20,36]. Research and clinical results have demonstrated that polyethylene terephthalate is a stable, biologically inert compound that is safe for use in humans, as it has been used for decades in other medical devices such as surgical sutures, mesh and vascular grafts [37–41]. In 2004 pillar implants received US FDA clearance to treat snoring and obstructive sleep apnea [42].

This study aims to evaluate pillar implants’ efficacy in reducing socially significant snoring and, to a lesser extent, daytime sleepiness, and to determine the statistical significance of improvement in snoring frequency, loudness and daytime sleepiness during follow-up intervals. Because the patients are unaware of snoring, their sleep partners’ evaluation is mandatory.

## Materials & methods

### Study design & population sample

We conducted a prospective follow-up study in 30 consecutive adult patients who presented to the otolaryngology department at our institution with a primary complaint of socially unacceptable snoring, either with or without daytime sleepiness, and who underwent pillar implantation to manage their complaints. All patients were operated on by the same team at the same institution.

Inclusion criteria were: patients aged 18 years or oldere who had symptomatic social snoring due to palatal flutter based on physical exam, no previous clinical diagnosis of obstructive sleep apnea, no prior surgical treatment for snoring, presence of a bed/sleep partner who was willing to participate in the subjective evaluation of their snoring, and willingness to provide written informed consent. Exclusion criteria included patients with soft palate length less than 25 mm, morbidly obese patients with BMI ≥40 kg/cm^2^, patients who had a significant change in BMI during the follow-up intervals, and patients with a history of nasopharyngeal surgeries for snoring or tongue base obstruction (Friedman tongue position grades III or IV). Other patients excluded from this study were: pregnant or breastfeeding women, patients who had nasal polyposis, tonsillar hypertrophy (tonsil size grade ≥3), hypertrophic inferior turbinates, adenoid hypertrophy, retrognathia, craniofacial abnormalities or significant nasal septal deflection as determined on physical examination, those without a bed partner and patients with neurological, psychiatric, cardiovascular or bleeding tendency diseases. Patients who dropped out during follow-up sessions were also excluded from the study.

After detailed history was taken from the patients and their sleep partners, all patients underwent ear, nose, throat, head and neck physical examinations, fiber-optic nasopharyngoscopy and assessments of Friedman tongue position and BMI. Assessments of the nasal airway, tonsils size, uvular dimensions, Mallampati score, laryngoscopy grade and tongue base were also conducted. Patients with high risk for OSA by the physician assessment were excluded from the study unless polysomnography demonstrated the absence of OSA.

### Outcome measures

The primary outcome measures were assessing snoring frequency per week and snoring loudness using a ten-point visual analog scale (VAS), in which 0 represented no symptoms and 10 represented the most severe symptoms as determined by the patients’ bed partners [17,43]. The snoring frequency was measured by asking the patients’ sleep partners to report the number of days per week on which the patient had symptoms; partners were also asked to assess the average severity of snoring loudness using VAS over the study time. The secondary outcome measure was the daytime sleepiness assessment reported by the participants using a validated Arabic version of ESS, ranging from 0 to 24. ESS scores of 0–9 were considered normal, while scores of 10–24 indicated a high risk for daytime sleepiness [28,44,45]. All outcome measures were assessed preoperatively and at 1, 3, 6 and 12 months postoperatively. Subjective improvement was defined as decreasing snoring frequency or scale scores at follow-up intervals relative to the baseline, with or without statistical significance.

### Pillar implants procedure

**Figure 1.**
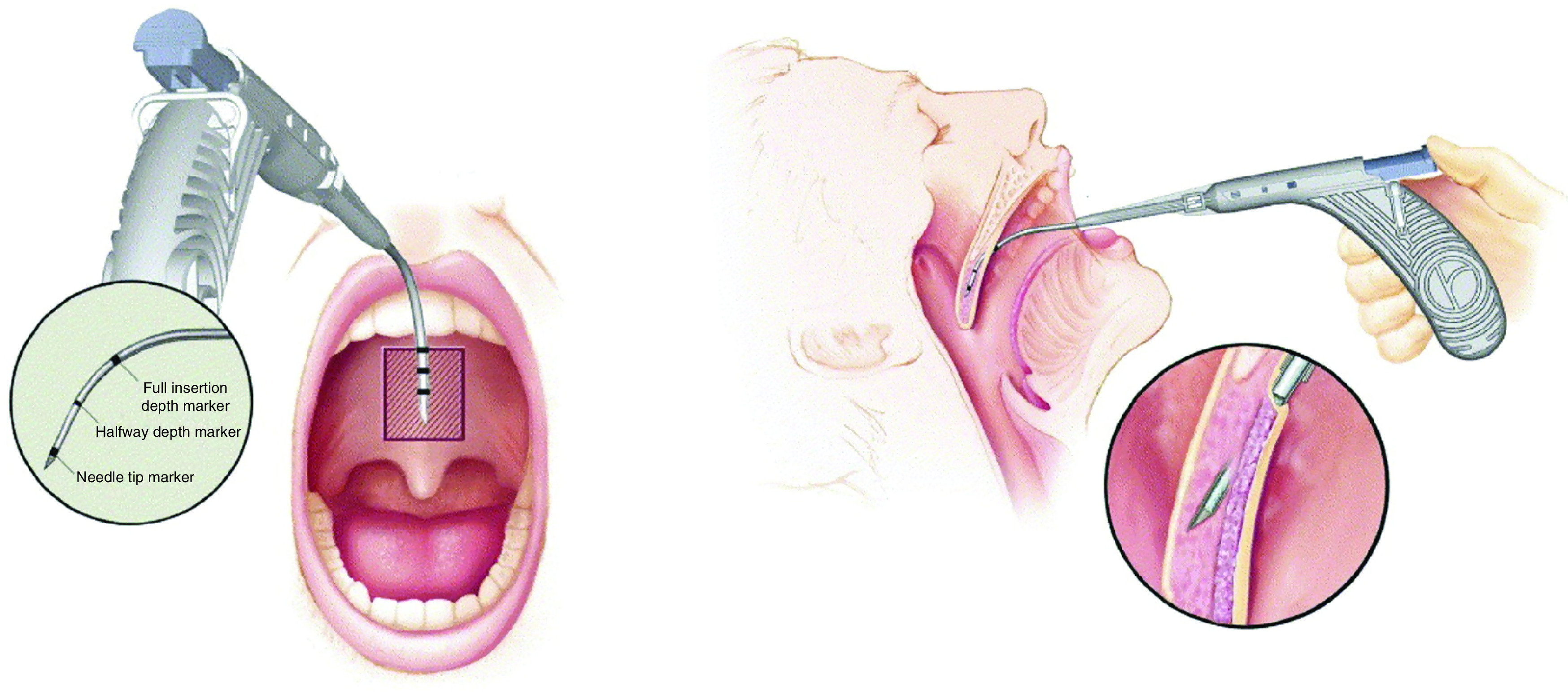
Pillar implant system procedure. There are three markers on the delivery handpiece: full insertion marker, halfway depth marker and needle tip marker. The handpiece is entering into the soft palate muscle, just below the hard palate. The needle is passed until the full insertion marker is reached. Modified with permission from [46] © Elsevier (2006).

**Figure 2.**
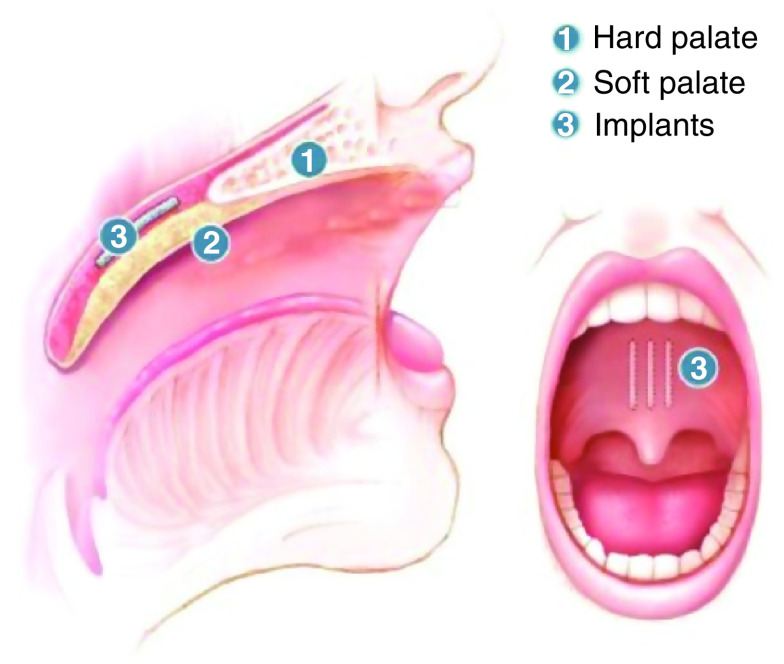
Implants are in the soft palate’s muscle layer, just below the junction of the hard and soft palates. The three implants are placed parallel to each other, 2 mm apart. Modified with permission from [46] © Elsevier (2006).

All patients received three separate pillar implants into the soft palate (Medtronics Inc., MN, USA). The cost of the procedure was about $600. The procedure was performed in the office using local anesthesia. Topical anesthetic spray and infiltration with 2 ml of a mixture of 1% lidocaine with epinephrine (1:100,000) were used to anesthetize the treatment area. Nasal decongestant sprays were applied to both nasal passages. Once local anesthetic had taken effect, the first implant was placed in the midline of the soft palate and the other implants were placed 2 mm to each side of the midline. The three implants were placed near the junction of the hard and soft palates, which is palpated with a tongue blade. A flexible nasopharyngoscopy was performed to ensure the correct positions of implants in the soft palate. If an implant is partially extruded, it must be removed in the clinical setting. At this point, the implant is not adherent to tissue and is easily removed; however, the wound was allowed to heal, and the implant was replaced if clinically indicated. Each patient received a 5-day course of broad-spectrum antibiotics following the procedure. Pain killers such as acetaminophen and nonsteroidal anti-inflammatory drugs were offered to be used as needed. No steroids were used during or after the procedure. A soft diet and oral rinses were recommended for 3 days after the procedure. Figures 1 & 2 represent the pillar implant procedure, as adapted from Walker *et al.* [46].

### Statistical analysis

The data were analyzed using SPSS v22 (IBM Corp., NY, USA). Patient characteristics were described using frequency and percentage for categorical variables and mean ± SD for continuous variables. Preoperative and postoperative mean values were compared using the paired Student *t* test. Statistical significance was reached with a p-value of < 0.05.

## Results

A total of 30 consecutive pillar implant patients (4 females and 26 males) were included in this study (Table 1). The patients’ average age was 44.7 years (range: 28–56; SD: 9.4), and the average BMI was 30.8 kg/m^2^ (range: 24.5–39.8; SD: 4.1). Nineteen patients were nonsmokers, while 11 smoked an average of one pack per day. Thirteen patients had undergone sinonasal surgery (endoscopic sinus surgery, septoplasty, turbinoplasty or a combination) at least 1 year before pillar implant. However, no nasal or oropharyngeal procedures were combined with the pillar implant. All the patients were followed up for 1 year.

**Table 1.** Demographic and clinical characteristics of included patients.

Variable	
Age (years), mean (±SD)	44.7 (±9.4)
Gender, frequency (%)	
Male	26 (86.7%)
Female	4 (13.3%)
BMI (kg/m^2^), mean (±SD)	30.8 (±4.1)
ESS score at baseline, mean (±SD)	7.4 (±5.4)
ESS score >10	10 (33.3%)
Partial implant extrusion, frequency (%)	2 (6.7%)
Follow-up time (months)	12

ESS: Epworth sleepiness scale.

Partial implant extrusion of one rod was seen in two patients (6.7%), and they underwent replacement surgery under general anesthesia. One patient experienced mild foreign body sensation and dysphagia in the first month post-operation, but these symptoms improved subsequently. One patient had odynophagia for 3 days after the operation, but it was reduced after treatment with a nonsteroidal anti-inflammatory drug.

### Frequency of snoring per week

At baseline, all patients complained of snoring throughout the week (7 days out of 7), except for one who reported snoring 4 days per week. The mean frequency of snoring per week decreased over the study period, from 6.9 before the operation to 6.1, 5.8, 5.5 and 5.0 at 1, 3, 6 and 12 months post-operation, respectively. Subjective improvements in snoring frequency by sleep partners were noted in 16.7, 26.7, 33.3 and 36.7% of patients at 1, 3, 6 and 12 months post-operation relative to the baseline, respectively.

There was a statistical difference between the mean preoperative snoring frequency and the frequency reported at 1, 3, 6 and 12 months post-operation (all p < 0.03). A statistically significant difference in the mean snoring frequency at 3 and 12 months (p < 0.03) was observed. However, the mean snoring frequency at other follow-up intervals was not statistically significant (1 vs 3 months, 3 vs 6 months, 6 vs 12 months: all p > 0.09).

### Snoring loudness

The mean VAS score for snoring loudness dropped from 9.2 before surgery to 7.2, 6.3, 6.1 and 5.9 at 1, 3, 6 and 12 months post-operation, respectively. Additionally, subjective improvements in VAS scores at 1, 3, 6 and 12 months post-operation were seen in 50, 73.3, 80 and 73.3% of patients, respectively, relative to the baseline.

The difference in loudness was statistically different from the preoperative visit at the 1-, 3-, 6- and 12-month visits (all p < 0.001). However, there were no statistical differences between the means of the postoperative intervals (all p > 0.07).

### Epworth sleepiness scale

**Figure 3.**
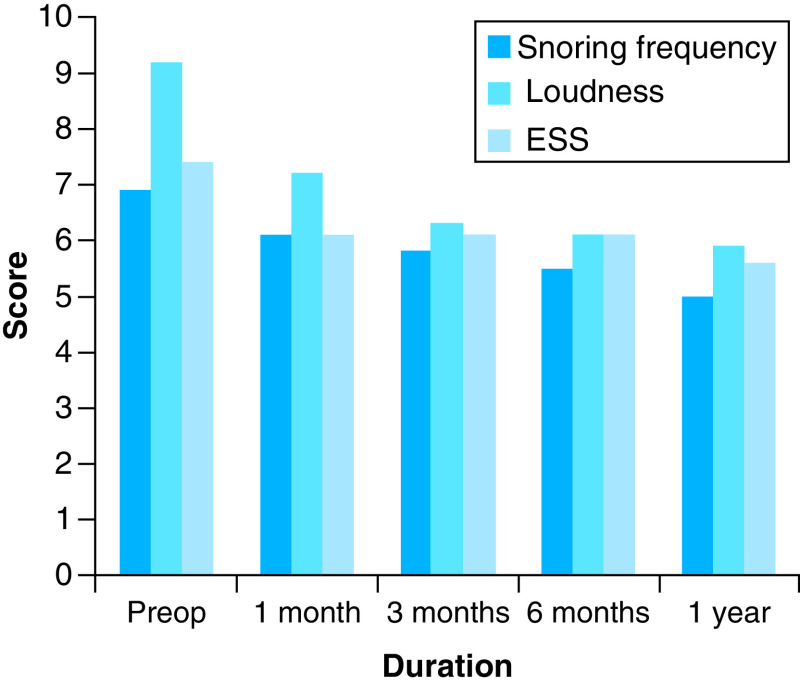
Changes in the means of scales through the follow-up intervals and relative to the baseline. ESS: Epworth sleepiness scale.

The ESS mean score decreased from 7.4 before the operation to 6.1 at the first month postoperatively, and this reduction remained stable at 3 and 6 months. At the 12th month post-operation, the ESS mean score decreased to 5.6, a statistically significant difference from the baseline (p < 0.01). The daytime sleepiness improved in 16.7, 26.7, 20 and 33.3% of patients at 1, 3, 6 and 12 months post-operation, respectively. Figure 3 demonstrates the mean scores of snoring frequency, loudness and daytime sleepiness at baseline and through the follow-up intervals.

On excluding patients who had ESS scores <10, only ten patients (33.3%) with excessive daytime sleepiness (ESS score >10) were included. The statistical analysis was repeated. It showed a significant difference in preoperative mean ESS score compared with that at 1 and 12 months post-operation (all p < 0.05).

## Discussion

This study demonstrates the high efficacy of the pillar implant technique in managing snoring and daytime sleepiness over a short period with a low complication rate. Pillar implants result in significant improvements of snoring frequency per week, VAS scores for snoring loudness, and daytime sleepiness using ESS scores at 1, 3, 6 and 12 months post-operation. The partial implant extrusion rate was 6.7%.

The soft palate is the most vibrating part that results in snoring; therefore most surgeries are directed at stiffening the tissue to reduce vibration [17,20,21]. Pillar implants cause a chronic inflammatory reaction that forms a fibrous capsule around the implants, resulting in scarring and stiffening of the soft palate and preventing its fluttering [17,46]. Other surgical treatment options for snoring are often associated with morbidity and short-term improvement [47]. Since the first use of pillar implants in early 2000, many studies have shown their efficacy in treating snoring and mild to moderate OSA [21,36,48]. The procedure has long-lasting effects with biologically compatible materials and fewer complications (e.g., pain and foreign body sensation) than are observed in other procedures such as UPPP, LAUP or radiofrequency thermal ablation (RFTA) [46]. Because no tissue is physically removed, significantly less pain should be encountered with palatal implants than with a LAUP procedure. Radiofrequency treatments to the soft palate act to diminish snoring through a similar scar formation method, but unlike palatal implants, the effects of radiofrequency energy are delayed. Additionally, RFTA often requires more than one treatment, and its effectiveness may not be long-lasting, whereas the palatal implant procedure is single-stage, permanent and more effective in controlling snoring [49,50].

We observed subjective improvements after 1 year for 36.7 and 73.3% of patients in snoring frequency and loudness, respectively, and for 33.3% of patients in daytime sleepiness. These improvements were statistically significant compared with preoperative values and concordant with previous studies that found a significant improvement in snoring levels and daytime sleepiness on treatment with pillar implants [17,20,21,35,36,48,51,52]. Murer *et al.* found that snoring and daytime sleepiness reduced after pillar implantation, and this improvement remained unchanged over a year [20]. Moreover, Friedman *et al*. found subjective improvements of 79.3 and 51.7% in snoring and ESS, respectively, in 29 patients after a follow-up period of 7–12 months after pillar implantation [52].

In a short-term follow-up of 3 months, Nordgard *et al.* reported a decrease in VAS from 9.7 to 5.5 for ESS score and from 8.4 to 4.3 for snoring (both p < 0.001) [21]. Sezen *et al.* evaluated the efficacy of palatal implants for the treatment of snoring in 17 patients with primary snoring and found that ESS mean scores and mean VAS snoring, apnea and daytime sleepiness scores were significantly improved after 90 days following the operation [53]. A systemic review and meta-analysis conducted by Choi *et al.* found a significant reduction in ESS score and apnea–hypopnea index (p < 0.001) after pillar implantation, with a mean extrusion rate of 9.3% [48]. Our findings are consistent with these results.

Saylam *et al.* found that subjective improvement in snoring in long-term follow-up (18 months) was 52.3%, and the mean ESS scores decreased from 7.8 to 5.5 after 6 months [18]. The authors did not find any difference between the results at 6 months, 1 year and 18 months, and concluded that the results at month 3 predicted the long-term benefits. However, in our study, we found that snoring frequency and loudness statistically improved at 1 month post-operation and continued to improve over the follow-up time of 12 months. Our finding was supported by the fact that fibrous capsule formation around the pillar implant is usually complete within 4 weeks [18]; therefore we believe that the results in month 1 could predict the long-term benefits.

There are many explanations for our nonresponding patients. High BMI (>40 kg/m^2^) could be one of the causes; however, Choi *et al.* and Friedman *et al.* found no effect of BMI on success rate [48,52]. Three implants were not enough to accomplish soft palate stiffness in some patients, and more implants may be needed; however, the soft palate becomes thinner more laterally, and the possibility of implant extrusion increases. Other reasons for snoring and daytime sleepiness could explain the nonresponders; for example, tongue base-associated obstruction might be misdiagnosed in the clinic, because the tongue position while the patient is awake is not the same as that during sleep [21]. Such patients could benefit from staged nasal and/or oropharyngeal procedures. Coexisting OSA might be another cause for nonresponding subjects. Polysomnography is mandatory to rule out such a condition, but the high cost and limited availability restrict its use.

Our findings support the suggestion of Nordgard *et al.* that management of snoring and daytime sleepiness by pillar implantation is a reasonable first-line approach due to its many advantages over the other more aggressive procedures [21]. Some of these advantages include its being office based, simple, single staged, usually performed under local anesthesia, tissue sparing and reversible without consequences, as well as its negligible associated discomfort and low complication rate. The cost of CPAP or surgery as treatment options for snoring and OSA is a primary concern, especially in developing countries. The pillar implant procedure cost was $600, which could be cheaper than CPAP and other treatment options. It is also possible to increase the palatal stiffness by inserting more implants in the soft palate.

The pillar implant procedure for snoring treatment invites comparison to other techniques. A direct comparison cannot be made here, as we have not performed a head-to-head trial of different techniques. However, the literature should be reviewed so that adequate counseling can be provided to patients regarding various surgical alternatives. Other common surgical techniques specifically for snoring include LAUP, RFTA and UPPP. LAUP is an outpatient surgical procedure for snoring treatment, including removing redundant palatal tissue and creating scarring of the soft palate to reduce snoring. Previous studies have suggested that its success rate may decrease over time, and its complications include pain, bleeding, candidiasis, local infection, velopharyngeal insufficiency, globus sensation, dryness, temporary palatal incompetence and temporary loss of taste or smell [54–56]. RFTA is also an outpatient procedure, in which a shielded needle is placed in various soft palate areas and radiofrequency energy is delivered submucosally, eventually resulting in soft palate scarring and decreased palatal flutter and snoring. Its success rates may also decrease over time [49]. Postprocedural complications include occult mucosal injuries, pain, speech deficits and dysphagia [57,58].

UPPP is an invasive procedure that includes expanding the retropalatal space by removing the free edge of the palate, the uvula, tonsils, adenoids and excessive oropharyngeal mucosa. Its long-term efficacy is low and it may cause many complications [59–61]. A systemic review and meta-analysis conducted by Franklin *et al.* suggested persistent complications occurred after UPPP and uvulopalatoplasty in about half the patients, of which swallowing difficulty, globus sensation and voice changes were the most common [59]. Severe complications were also reported, including death, bleeding, infections, cardiac arrest and respiratory compromise. The authors suggested that the evidence of harm was more significant than the evidence of benefits in such procedures [59].

There are new minimally invasive maneuvers for snoring and OSA treatment, including suspension sutures and modified anterior palatoplasty (MAP). In 2016 El-Ahl and El-Anwar presented the single suspension suture (SSS) to ensure anterolateral advancement of the palate and lateral pharyngeal wall [62]. The SSS procedure included a rounded needle with Vicryl passed through the palatopharyngeus muscle and palatal muscles toward the pterygoid hamulus, then returned near the start point in the tonsillar bed. In December 2017, Askar and El-Anwar described a double suspension suture which involved, besides the SSS, a second suture between the palatopharyngeus muscle in the tonsillar bed to the pterygomandibular raphe, through the soft palate and then returned to the start point at the palatopharyngeus muscle [63]. Both techniques were proved to be simple, fast, rapid, effective and of low cost, with minimal complications [62–64]. However, their efficacy is limited for treating a retropalatal obstruction in snoring or OSA patients [64,65].

Anterior palatoplasty is a modified technique that involves removing a horizontal trapezoid strip of soft palate mucosa. The mucosa and submucosal tissues are then stripped down to the muscular layer using electrocautery and then the stripped area is sutured, with three sutures passed in the soft palate to include mucosa, submucosa and muscles in a multistation, multilayer fashion, widening the retropalatal area to reduce snoring. A systematic review by Pang *et al.* found that the success rate of anterior palatoplasty reached 72.5% with a mean follow-up of 17.3 months, accompanied by significant improvements in the apnea–hypopnea index, snoring VAS scores and ESS scores, with no complications except for pain [66]. MAP is a simple, quick, inexpensive, office-based, single-stage procedure associated with excellent outcomes and minimal complications and useful for simple snorers and patients with mild to moderate OSA [67,68]. Askar *et al.* suggested combining MAP and double suspension suture for better outcomes in OSA patients with predominant retropalatal obstruction [69].

Based on our findings and a literature review of surgical treatment options for snoring, we suggest that pillar implants are comparable to suspension sutures and MAP in terms of simplicity, efficacy and low complication rate in treating snoring, while they are superior to LAUP, RFTA and UPPP in such terms.

However, our study had certain limitations. First, an obvious limitation was the small study size. Also, the study was not placebo-controlled; it is challenging to conduct such trials for surgical procedures because sham surgery is not acceptable. Additionally, for financial reasons a sleep study (polysomnography) was not conducted for all patients in our study as it is not necessary to diagnose snoring and daytime sleepiness; therefore coexisting OSA and silent apnea, which might cause nonresponding subjects, could not be ruled out even when subjective improvements were reported. We did not select cases with specific sites or patterns of upper airway obstruction/collapse; rather, we only performed the surgery on various cases with socially unacceptable snoring complaints. Askar *et al.* suggested that preoperative topographical diagnosis of OSA using positional awake endoscopy with Müller’s maneuver while the patient is in a supine position (preoperative endoscopic examination of the upper airways) allows for tailored surgical management and could yield better surgical outcomes because the technique is chosen based on the preoperative topographical assessment [70]. Selection of our patients was based totally on clinical examination, and the results were dependent on the subjective opinions of the patients and their sleep partners. Due to the nature of the study design and lack of a control group, it is difficult to draw any definitive conclusions, especially considering the selection bias and possible placebo effects that affect subjective outcomes. Another limitation is the relatively short follow-up period which was 12 months; the results might differ in a longer follow-up. Rotenberg *et al.* conducted follow-up for the longest time (4 years) and found that snoring improvement after the first year declined by 50% over the next 3 years [35]. However, the improvement after 4 years was still significant compared with preoperative values, and the authors still recommended pillar implants as a safe and effective procedure with a low complication rate.

## Conclusion

Pillar implantation is a safe and effective office-based procedure with a low complication rate. In our study it significantly improved snoring and daytime sleepiness over 1 year. We recommend this procedure for managing snoring and daytime sleepiness in selected patients before proceeding to more aggressive surgeries. Our study showed that the results after 1 month post-operation could predict the long-term benefits for snoring frequency and loudness. Although subjective improvement was observed in daytime sleepiness at 1 month post-implantation, the clinical significance will be apparent after 1 year.

## Future perspective

With the findings of this study, providers should be aware of pillar implantation’s effectiveness in controlling snoring and daytime sleepiness symptoms and thus improving sleep-related quality of life in such patients. As it is a minimally invasive, simple, office-based procedure with a low complication rate, we advise it before proceeding to more morbid surgeries, especially in patients who failed or refused CPAP treatment. More extensive studies with longer follow-up are needed to predict its long-term efficacy.

**Summary points**

- Snoring and daytime sleepiness are common sleep-related problems worldwide that negatively impact performance, health and safety and cause social embarrassment and sleep-partner disharmony and discord. Therefore proper management of excessive daytime sleepiness is of paramount importance to improve quality of life.
- We assessed the efficacy of pillar implants in reducing snoring and, to a lesser extent, daytime sleepiness over 1 year after implantation. Improvements were measured using sleep partners’ assessments of snoring frequency per week, a visual analog scale (VAS) for snoring loudness, and patients’ Epworth sleepiness scale (ESS) scores for daytime sleepiness.
- A total of 30 consecutive pillar implant patients (4 females and 26 males) were included in this study. They were assessed preoperatively and at 1, 3, 6 and 12 months after the implantation.
- The pillar implant procedure resulted in significant snoring frequency improvements and reduced VAS scores and ESS scores at 1, 3, 6 and 12 months post-operation.
- The mean frequency of snoring per week dropped from 6.9 before the operation to 6.1, 5.8, 5.5 and 5.0 at 1, 3, 6 and 12 months post-operation, respectively (all p < 0.03).
- The mean VAS score for snoring loudness decreased from the baseline of 9.2 before surgery to 7.2, 6.3, 6.1 and 5.9 at 1, 3, 6 and 12 months post-operation, respectively (all p < 0.001).
- The mean ESS score decreased from 7.4 before the operation to 6.1 at 1 month post-operation and 5.6 at 12 months post-operation (p < 0.01).
- Partial implant extrusion was seen in only two patients (6.7%).
- In conclusion, pillar implantation is a quick and simple office-based procedure with minimal morbidity that could significantly improve sleep-related quality of life in patients complaining of snoring and/or daytime sleepiness.
